# Technology use and attitudes towards digital mental health in people with severe mental health problems: a survey study in China

**DOI:** 10.3389/fpsyt.2023.1261795

**Published:** 2023-11-22

**Authors:** Xiaolong Zhang, Shôn Lewis, Xu Chen, Natalie Berry, Sandra Bucci

**Affiliations:** ^1^Division of Psychology and Mental Health, School of Health Sciences, Faculty of Biology, Medicine and Health, Manchester Academic Health Science Centre, The University of Manchester, Manchester, United Kingdom; ^2^The National Clinical Research Center for Mental Disorders and Beijing Key Laboratory of Mental Disorders, Beijing Anding Hospital, Capital Medical University, Beijing, China; ^3^Greater Manchester Mental Health NHS Foundation Trust, Manchester, United Kingdom; ^4^Advanced Innovation Center for Human Brain Protection, Capital Medical University, Beijing, China

**Keywords:** implementation, psychosis, bipolar disorder, major depressive disorder, COVID-19, smartphone

## Abstract

**Introduction:**

Digital mental health is a promising solution to support people with severe mental health problems (SMI) in China. However, little is known about the ownership rate of digital technologies and attitudes towards utilising digital health technologies (DHTs) among people with SMI in the Chinese context. The aims of this study were to understand: (i) digital technology ownership and usage rate of people with SMI in China; (ii) attitudes toward DHTs in mental health services; and (iii) how the COVID-19 pandemic has influenced views on digital mental health.

**Methods:**

A cross-sectional survey was given to outpatients with SMI using the REDCap platform. To capture a diverse sample of people with SMI, the survey was distributed across psychiatric hospitals, general hospitals with a psychiatric unit, secondary hospitals, and community healthcare centres.

**Results:**

In total, 447 survey respondents completed the survey. Relative high ownership rates of digital technologies were found, with smartphone ownership (95.5%) and access to the internet (82.1%) being the highest technologies reported. However, less than half of respondents reported frequent health-related usage of digital technologies, which may be related to the lack of knowledge in using DHTs. Most respondents found DHTs being useful for access to mental health services during the pandemic and were willing to use DHTs after the pandemic.

**Discussion:**

Our data suggest that, despite the high ownership rate of digital technologies, training programmes to improve digital health literacy for people with SMI in China are necessary to realise the full potential of digital mental health.

## 1 Introduction

Severe mental health problems (SMI; i.e. schizophrenia and other psychotic disorders, bipolar disorder, and major depressive disorder) can cause significant functional impairment and burden (1). People with SMI face excess mortality reflected by 10 to 20 years shorter life expectancy compared to the general population (2). In China, mental health problems accounted for 20.29 million disability-adjusted life years (DALYs) in 2019, and depressive disorders and schizophrenia were the leading causes, accounting for 29.03 and 13.70% of DALYs of mental and substance use disorders, respectively (3). According to a Chinese mental health survey, the lifetime prevalence of schizophrenia or any other psychotic disorder, bipolar disorder, and major depressive disorder in China was 0.7, 0.6, and 3.4%, respectively (4). Moreover, nearly 40% of people with mental health problems were living in poverty (5), and the rate of poverty in people with SMI increased 9.5% between 1994 and 2015 in rural China (6).

Timely access to mental healthcare is crucial for the recovery of people with SMI; however, due to the shortage of mental health professionals (7, 8) and a hospital-centred healthcare system (9), China’s mental health services struggle to meet the treatment needs of people with SMI, especially for people living in rural or remote areas (10, 11). China has initiated a series of reforms to improve the accessibility and quality of mental health services in recent decades (12). In 2004, the Chinese government launched the National Continuing Management and Intervention Programme for Psychoses (i.e., the 686 programme), which aims to integrate hospital and community-based mental health services for people with SMI (13). The programme monitors the treatment of community-dwelling individuals with SMI and offers free medications to those who cannot afford the services. Thus far, more than 6 million people with SMI have registered in the programme, and more than 70% of enrollees have received antipsychotic treatment (14). Building on the 686 programme, the National Information System for Psychosis was established in 2011 (15) and the National Comprehensive Management Pilot Project for Mental Health was built in 2015 (16). However, the gap in the access and quality of mental health services still exists, and disparity between economically developed areas and economically underdeveloped areas has increased (17). The effort to transfer most mental health services from psychiatric hospitals in urban areas to general hospitals and rural health clinics has only partially succeeded (18). Furthermore, the community-based services still needs to be strengthened, especially in western China (15). Hence, innovative approaches are warranted to optimise the management of SMI in China.

Given the rapid development of digital technologies in China, digital mental health may be relevant for managing SMI in China. The ownership rates of smartphone in China in the general population have risen to 96% (19). With the ubiquitous accessibility of digital technologies, digital mental health has the unprecedented potential to scale-up service provision. Digital health technologies (DHTs) can be used in a standalone manner or in combination with other health services such as medical devices, diagnostic tests, or face-to-face appointments (20). The advantages of DHTs compared to traditional mental health services are clear, such as improving accessibility to services, social interaction and peer support via online platforms, low cost, timely information sharing between health providers and service users, anonymity, and normalising mental health problems (21). Regarding the management of SMI more specifically, DHTs have the potential to improve access to healthcare (22), reduce cost for healthcare providers (23), enhance symptom monitoring (24, 25), and provide more timely and personalised intervention (26–28). In addition, digital phenotyping has the potential to inform diagnosis and personalised treatment (29). DHTs have been proliferating in China in the last few years. A recent systematic review on DHTs in China reported that 32 DHTs were developed and tested for a range of mental health problems in the past 5 years alone (30). Efficacy of DHTs targeting schizophrenia, depression, anxiety, substance use disorder, and trauma have been demonstrated by randomised controlled trials conducted in China. Meanwhile, on commercial app stores, 172 mental health-related apps for psychological counselling, mental health assessment, stress management, psychoeducation and multipurpose apps (i.e., a combination of counselling and assessment) were available for download (31).Despite the potential for DHTs to optimise the management of SMI, real-world uptake and adoption remain limited around the globe (32). Key barriers include ‘digital divide’ and the lack of understanding and integrating stakeholders’ views towards DHTs in the design and development process (33, 34). Nonetheless, little is known about the ownership rates of digital technologies among people with SMI in China and their views and attitudes toward utilising DHTs. To date, only two studies have been conducted to explore views of people with mental health problems in China on DHTs. One survey study investigated the views of people with mental health problems and their family members on mobile health interventions, and the study found that the most participants were willing to accept mobile health interventions and considered them helpful (35). However, this study included participants with a broad range of mental health problems, and the results of people with mental health problems were reported combined with their family members, making the opinions of people with SMI unclear. Another survey explored the habits and attitudes of video gaming and information technology usage in people with schizophrenia in Hong Kong (36). The study found that 90.9% of the participants had access to the internet and half of the sample used the internet daily; nevertheless, the acceptability of other types of DHTs was not investigated. Therefore, to ensure DHTs meet the needs of people with SMI, and to support the implementation of digital mental health in China, a comprehensive study on the views of people with SMI about digital mental health should be explored.

Furthermore, DHTs have been heavily relied on in clinical practice during the COVID-19 pandemic, given the significant disruption of mental healthcare provision caused by restrictions on in-person mental health services. For instance, 17 different countries have changed their regulations and increased utilisation of telemedicine for mental healthcare to mitigate the risk of spreading the virus and the disruption of healthcare provision (37). As has been seen in many countries, more hospitals in China are providing online telemedicine care after the outbreak of COVID-19 pandemic (38). As the pandemic has accelerated the implementation of DHTs worldwide, an investigation on the impact of the pandemic on views of people with SMI on DHTs is necessary to inform the implementation of DHTs in the post-COVID era.

Therefore, to address the aforementioned gaps, the current survey-based study explored: (i) digital technology ownership and usage rates of people with SMI in China; (ii) attitudes toward digital technology in mental health services; and (iii) how the COVID-19 pandemic has influenced views on digital mental health.

## 2 Methods

### 2.1 Participants

An online survey was conducted from February 2021 to January 2022 using the REDCap platform (39, 40). To reach a diverse sample of people with SMI, participants were recruited from multiple sites in Beijing, including a tertiary psychiatric hospital (i.e., Beijing Anding Hospital), psychiatric unit of two tertiary general hospitals (i.e., Beijing Anzhen Hospital and Beijing Chaoyang Hospital), two secondary hospitals (i.e., Pingan Hospital and Shunyi Hospital), and a community healthcare centre (i.e., Xinjiekou Community Healthcare Centre). These hospitals are members of the research network of the National Clinical Research Centre of Mental Disorders (NCRCMD) based in Beijing Anding Hospital. The NCRCMD research network is a nationwide clinical research collaboration organisation and has 75 membership hospitals across China. The recruitment advertisement was disseminated through the research network of NCRCMD to the co-ordinators of the seven membership hospitals abovementioned. Participants were selected based on the following eligibility criteria: (1) aged 18 years and older; (2) have a diagnosed severe mental health problem (i.e., schizophrenia, schizoaffective disorder, bipolar disorders, major depressive disorder); (3) fluent in speaking Mandarin Chinese; (4) able to provide informed consent. Exclusion criteria were: (1) not able to provide informed consent, as judged by a registered mental health professional; (2) at risk for self-harm or harm to others; (3) adults detained under mental health legislation. Eligibility criteria were verified by the referring clinicians based on the participant’s medical record. Potential participants were approached by their treating clinicians while visiting the outpatient clinic of the hospital. The URL and the QR code to access the online survey were given to potential participants if they agreed to take part.

The ethics committee of the University of Manchester and Beijing Anding Hospital of Capital Medical University approved the study. An electronic participant information sheet and consent form was embedded in the survey.

### 2.2 Survey design

The survey was developed based on previous studies (41, 42) and the prior work of our own research group. The survey was initially designed in English by the first author and then translated into Chinese. The English version was reviewed by the co-authors (SL and SB). The Chinese version was tested with six people with psychosis recruited from Beijing Anding Hospital to assess the readability and appropriateness of the survey. The survey was refined according to feedback. The final survey was disseminated on the REDCap platform. The English and Chinese versions of the survey are shown in Supplementary Tables 1 and 2, respectively.

The survey comprised four sections:

1. *Demographic information*: this section recorded basic demographic and clinical information, including, age, gender, current location, living arrangements, marital status, level of education, employment status, household income, and diagnosis of mental health problem.
2. *Ownership and usage of DHTs*: this section consisted of seven sets of multiple-choice questions about participants’ ownership of digital technologies (e.g., smartphone, computer, internet, etc.) and their experiences of using DHTs (i.e., usage of mental health related apps, barriers to own or use a phone, experiences of sharing information with health providers, and their interest in future digital mental health services), and two sets of seven-points Likert scale (1 = multiple times a day, 7 = less often) and one 5-point Likert scale (1 = very often, 5 = never) about the frequency of using DHTs and their features.
3. *Attitudes towards digital health interventions*: this section was adapted from the Attitudes towards Psychological Online Interventions Questionnaire [APOI; (43)]. The APOI assesses respondents’ acceptance of Internet interventions along four dimensions (Scepticism and Perception of Risks, Confidence in Effectiveness, Technologization Threat, and Anonymity Benefits) on a five-point Likert scale (1 = totally agree, 5 = totally disagree). We modified the items of the questionnaire to fit the aims of the current study. The total score of the scale ranges from 16 to 80, with a higher score indicates a more positive attitude.
4. *The impact of the COVID-19 pandemic*: this section comprised three 5-point Likert scales (1 = not at all, 5 = very much) assessing how respondents’ accessibility of mental healthcare and their views on digital mental health have been influenced by the COVID-19 pandemic.

### 2.3 Statistical analysis

Statistical analysis was performed using the R software package [version 4.0.5; (44)]. To summarize the digital technology ownership and usage rates among the respondents and the influence of the COVID-19 pandemic on views toward digital mental health, descriptive analyses, including frequencies and percentages, were performed on the data pertaining to these aspects. In order to understand respondents’ attitudes towards digital technology in mental health services, we conducted a linear regression analysis to explore the impact of specific demographic factors such as age, gender, education level, and diagnosis. Of note, the online survey platform used in this study required respondents to answer all the questions, ensuring that there was no missing data.

## 3 Results

### 3.1 Demographic characteristics

Total survey views was 934. Of these, 710 participants provided consent to take part in the study, and 473 completed the survey, resulting in a completion rate of 50.6% (*n* = 473/934). 26 of the completers were excluded from analysis for not meeting the eligibility criteria (i.e., age less than 18 years). Data from 447 participants was analysed.

Demographic information is shown in Table 1. Most respondents were female (*n* = 346/447, 77.4%), with a mean age of 34.8 years (SD = 14.3; range 18–92). Most respondents had a diagnosis of MDD (*n* = 208/447, 46.5%), and one fifth of the respondents had a diagnosis of a psychotic disorder (i.e., schizophrenia and schizoaffective disorder). Since recruitment was conducted only in Beijing, most participants were living in east China. Most respondents were single (*n* = 254/447, 56.8%), had university or some university education (*n* = 247/447, 55.3%). Furthermore, a significant portion of the respondents were working full-time (*n* = 160/447, 35.8%). Regarding income level, a considerable number of respondents received a 1,000 to 5,000 RMB (approximately USD 140 to 700) monthly income (*n* = 160/447, 35.8%), which is middle income according to the National Bureau of Statistics of China (45).

**Table 1:** Characteristics of survey respondents.

Characteristics	Values, *n* (%)
Gender	
Male	101(22.6)
Female	346(77.4)
Age group	
18–25	144(32.2)
26–30	68(15.2)
31–40	110(24.6)
41–50	58(13.0)
51–60	37(8.3)
>60	30(6.7)
Geographic location	
East China	413(92.4)
Central China	15(3.3)
West China	7(1.6)
Northeast China	12(2.7)
Living arrangements	
Living alone	60(13.4)
Living with others	387(86.6)
Marital status	
Single	254(56.8)
Married	160(35.8)
Divorced	28(6.3)
Widowed	5(1.1)
Highest level of education	
Postgraduate course	61(13.6)
University or some university	247(55.3)
High school or some high school	139(31.1)
Employment status	
Working full-time	160(35.8)
Working part-time	4(0.9)
Self-employed	61(13.7)
Student	107(23.9)
Currently unemployed	115(25.7)
Monthly household income per person	
Under 1,000 RMB	30(6.7)
1,000–5,000 RMB	160(35.8)
5,000–10,000 RMB	124(27.7)
10,000–15,000 RMB	61(13.7)
Above 15,000 RMB	72(16.1)
Diagnosis	
Schizophrenia	89(19.9)
Schizoaffective disorder	4(0.9)
Bipolar disorder	146(32.7)
Major depressive disorder	208(46.5)

### 3.2 Access to digital technology and digital health tools

**Figure 1:**
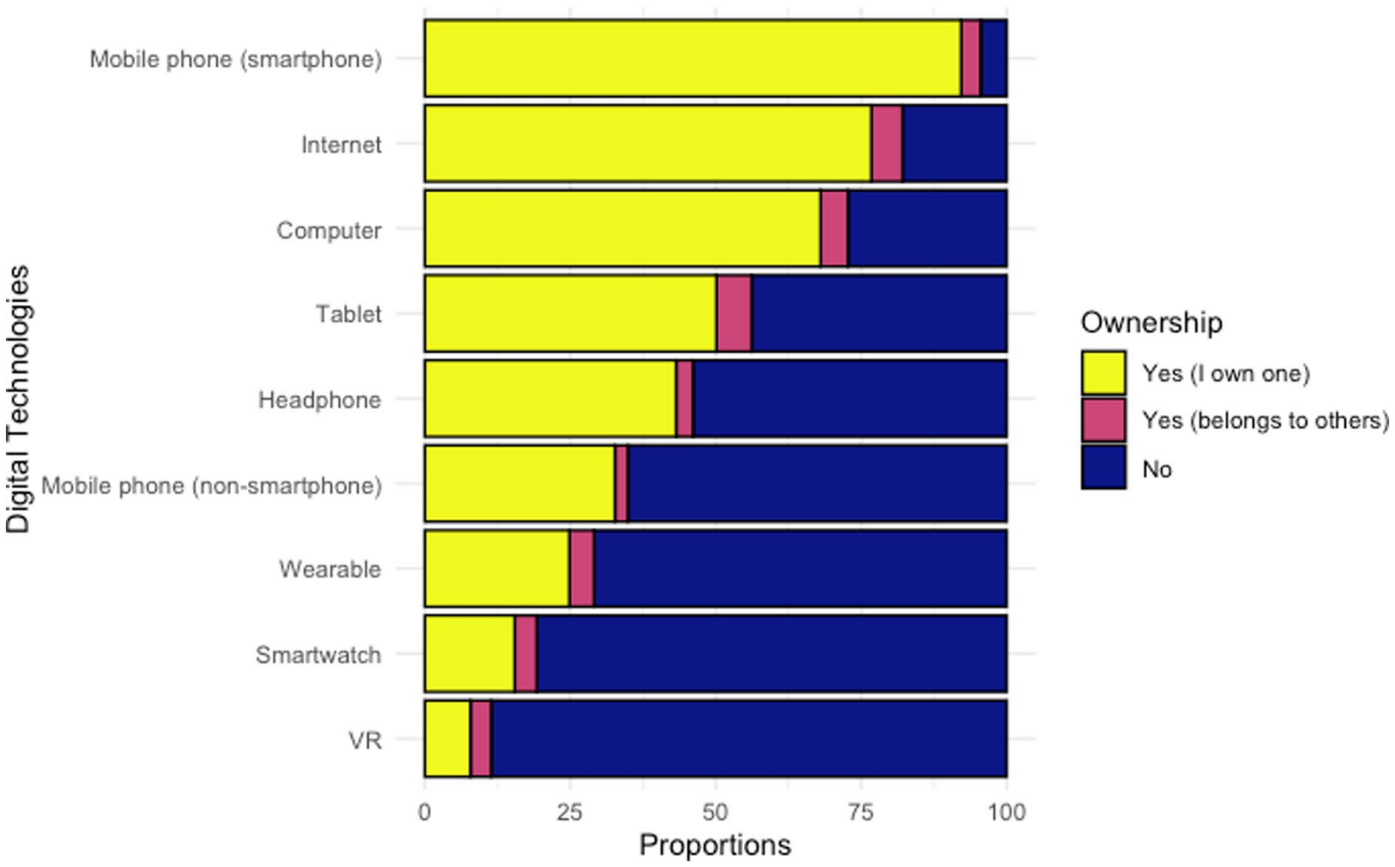
Ownership of digital technologies.

**Figure 2:**
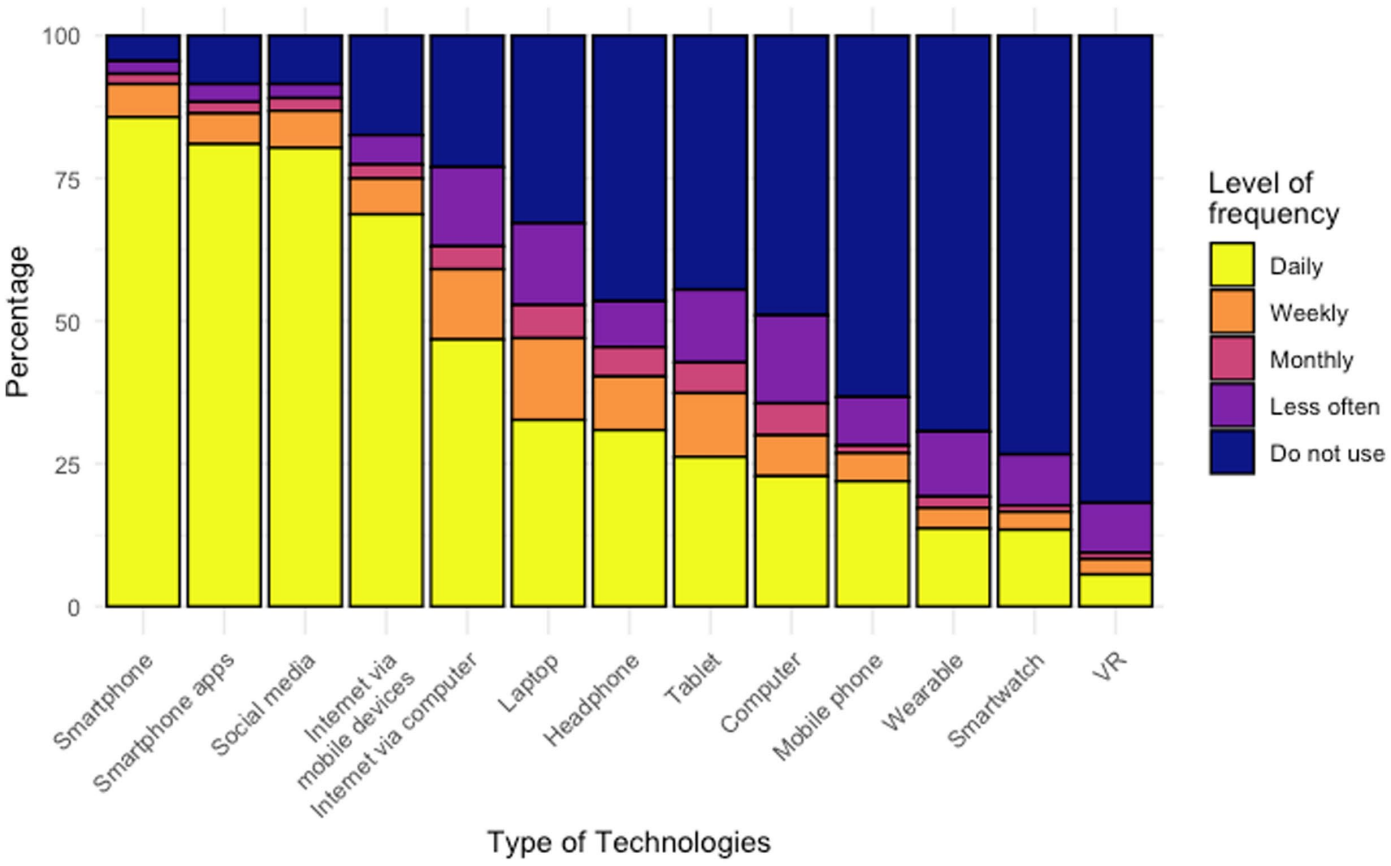
Frequency of using different digital technologies.

As shown in Figure 1, the digital technology most respondents had access to was a smartphone, with nearly all respondents (*n* = 427/447, 95.5%) reporting either owning a smartphone by themselves or having access to one that was owned by someone else, followed by internet (*n* = 367/447, 82.1%) and computer (*n* = 325/447, 72.7%). Only a small proportion of respondents had access to a wearable device (*n* = 130/447, 29.1%), smartwatch (*n* = 86/447, 19.2%) or VR (*n* = 51/447, 11.4%). The frequency of using digital technologies is displayed in Figure 2. Over three quarters of respondents reported using a smartphone, smartphone apps, and social media daily. Like the patterns of digital technologies ownership, wearable, smartwatch, and VR were the least frequently used. When asked about the barriers for owning or using a mobile phone, top reasons were ‘do not know how to use certain features’, ‘feel unsafe’, and ‘not interested’, as shown in Table 2. Of note, nearly two third of respondents endorsed having no barriers for owning or using a phone.

**Table 2:** Barriers faced to being able to own or use a mobile phone.

Barriers	Values, *n* (%)
I do not know how to use certain mobile phone features (e.g., smartphone apps)	93(20.8)
I feel paranoid or suspicious about mobile phones	42(9.4)
I’m not interested in mobile phones	31(6.9)
I do not know how to use a mobile phone	25(5.6)
I keep losing or damaging mobile phones	15(3.4)
I struggle to afford to own and/or use a mobile phone	11(2.5)
I do not need to use a mobile phone	10(2.2)
There are no barriers for me	284(63.5)

**Figure 3:**
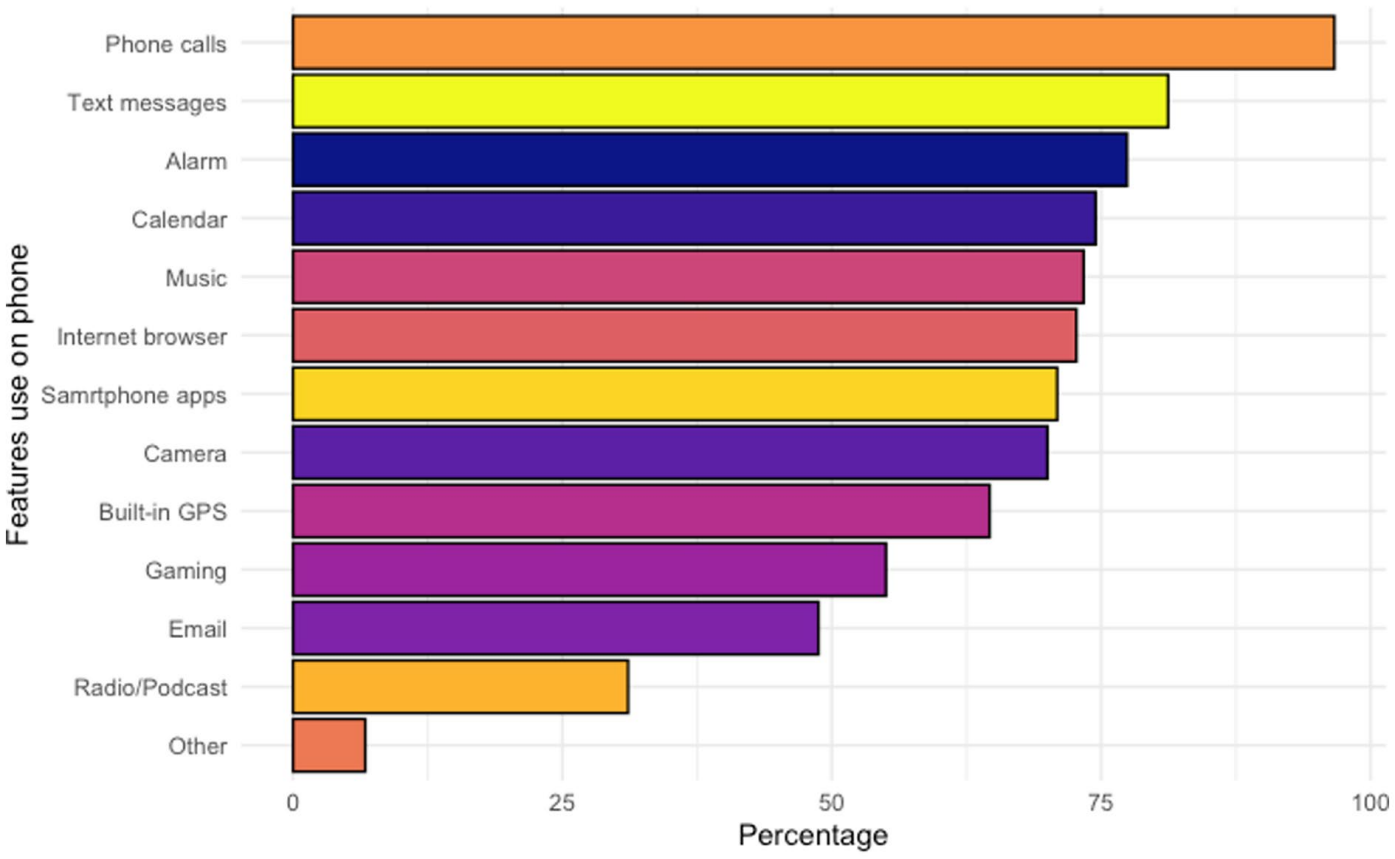
Features used on smartphone.

**Figure 4:**
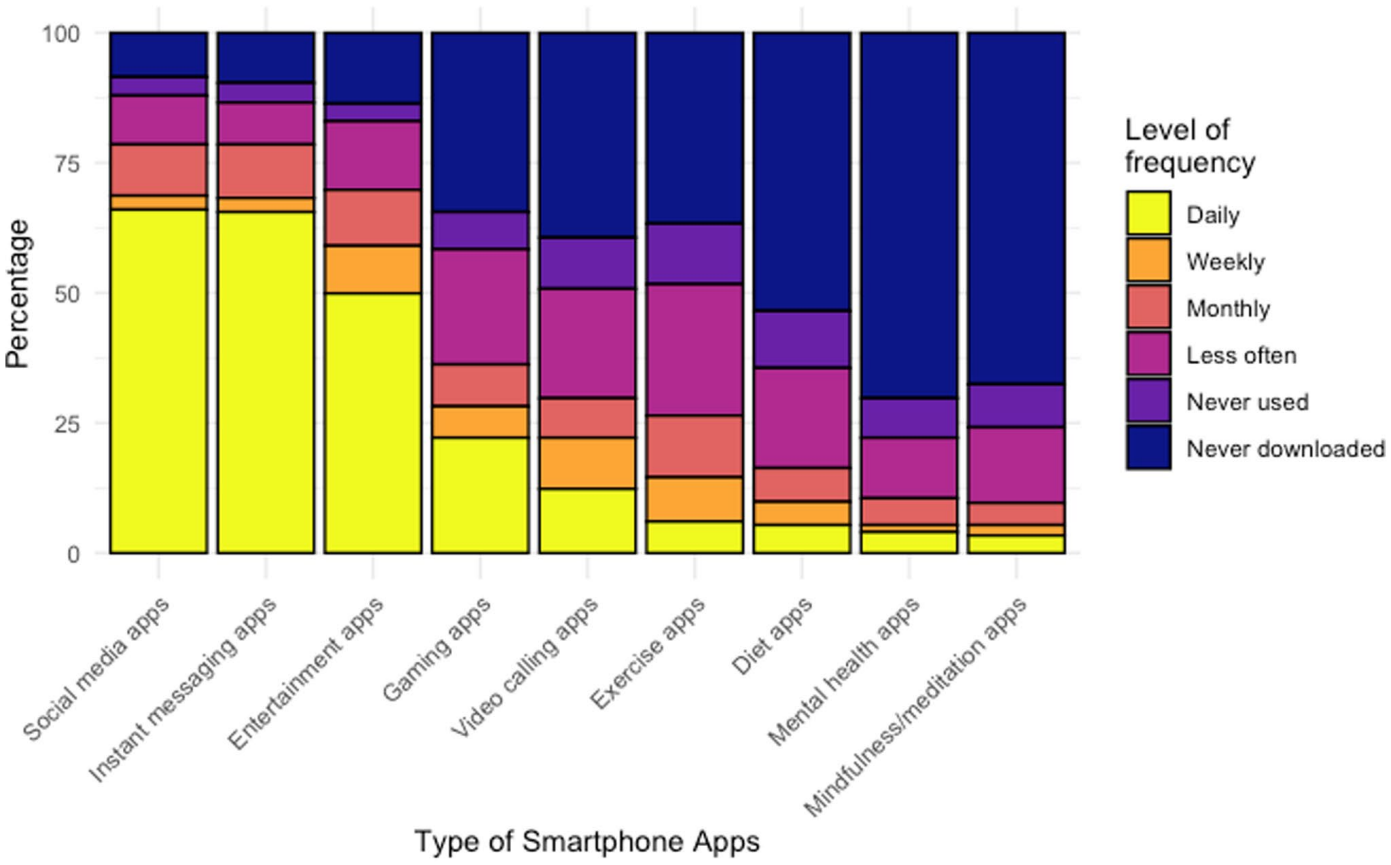
Frequency of different types of apps used on smartphone.

Regarding the operating system of smartphones, more than half of respondents (*n* = 251/447, 56.2%) were using an Android platform, whereas 37.8% (*n* = 169/447) respondents reported using iOS platform. Figures 3, 4 display the features and frequency of apps used on the phone. The most used features on phones were phone calls (*n* = 432/447, 96.6%), text messages (*n* = 363/447, 81.2%), and the alarm (*n* = 346/447, 77.4%). The most frequently used apps were social media apps, instant messaging apps, and entertainment apps, with the proportion of reported daily using being 66.0% (*n* = 295/447), 65.5% (*n* = 295/447), and 50.0% (*n* = 223/447), respectively. Regarding the usage of mental health-related apps, only 17 respondents reported 16 different apps that they had used or were using. Generally, respondents said these apps were helpful, but the frequency of use varied by apps. A detailed list of the apps reported is shown in Supplementary Table 3.

**Figure 5:**
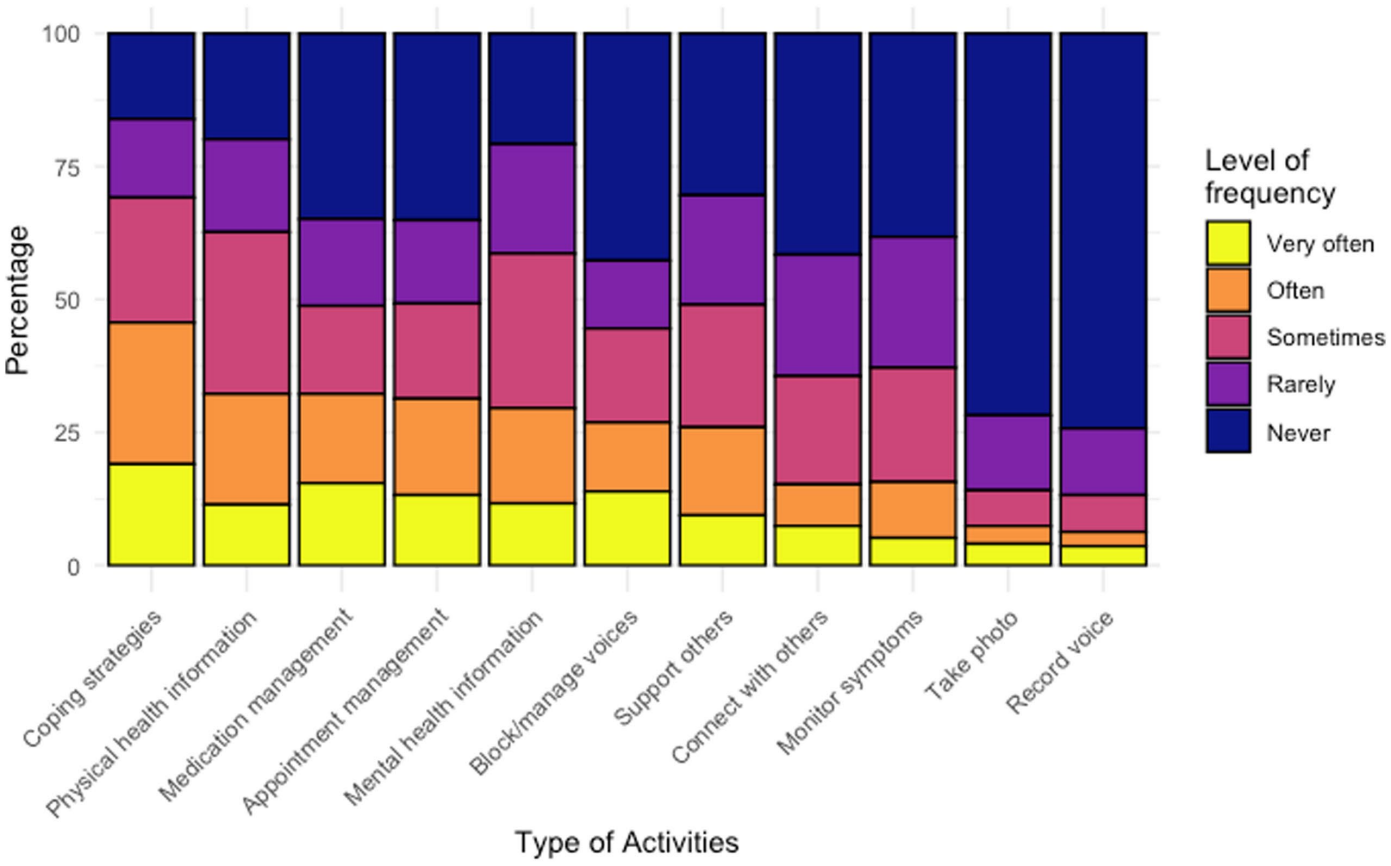
Frequency of health related usage of digital technologies.

The overall frequency of using digital technologies for health-related purposes was relatively low, with less than half of respondents reporting frequent usage for the activities shown in Figure 5. Finding coping strategies was the most endorsed activity, with nearly half of respondents (*n* = 204/447, 45.6%) describing ‘very often’ or ‘often’ doing so. However, less than half of the respondents reported having shared medication or psychotherapy information they found online with their care team, as shown in Table 3.

**Table 3:** Sharing information with mental healthcare team.

Items	Yes, *n* (%)	No, *n* (%)
Information found online about psychiatric medications	156(34.9)	291(65.1)
Information found online about psychological therapy	181(40.5)	266(59.5)

**Figure 6:**
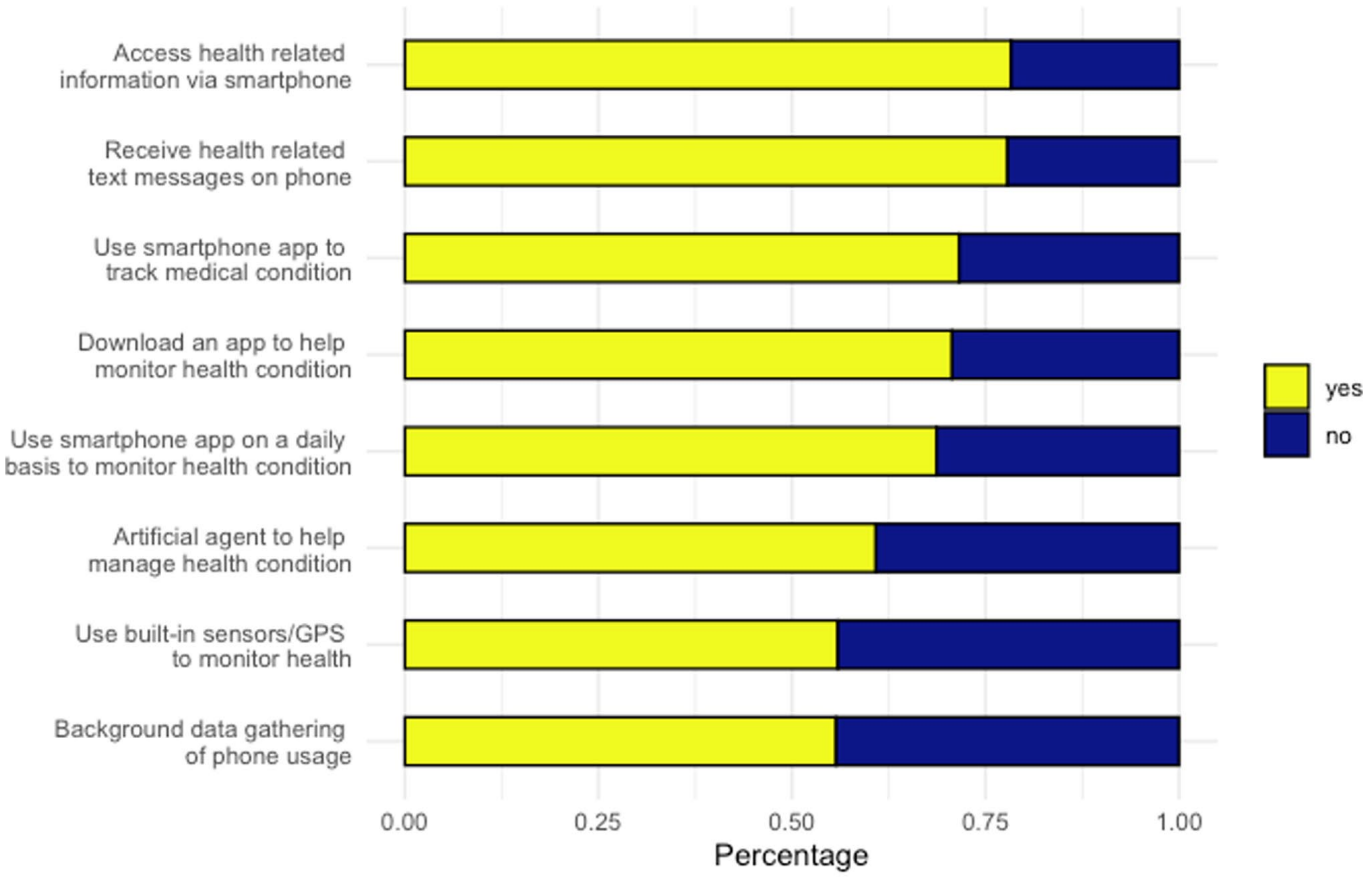
Interests in future digital health services.

Figure 6 shows respondents’ interests in future digital health services. Over three quarters of respondents endorsed interest in accessing general information (*n* = 350/447, 78.3%) and receiving text messages (*n* = 348/447, 77.9%) related to their health via a smartphone. However, features related to passive monitoring were less popular, with under half of respondents reporting willingness to permit symptom monitoring using background phone usage data (*n* = 249/447, 55.7%) or built-in sensors or GPS (*n* = 250/447, 55.9%).

### 3.3 Attitudes

Regarding attitudes towards digital health interventions, the average APOI total score was 48.2 (SD = 7.97), which indicated a neutral level of attitude of respondents. The results of linear regression analysis showed that age and education level significantly predicted APOI total score, with younger respondents reporting a higher APOI total score compared to older respondents (*p* < 0.001), and respondents with a University degree or higher reporting a higher APOI total score than respondents without a University degree (*p* < 0.01; Table 4).

**Table 4:** Linear regression analysis of demographic factors associated with attitudes towards digital health interventions.

Variables	Unstandardized coefficients, B (SE)	*t* test	*p* value
Age	−0.08 (0.03)	−2.77	< 0.001
Sex (reference: male)			
Female	0.99 (0.91)	1.09	0.27
Education level (reference: High school and below)			
University degree or higher	2.12 (0.87)	2.42	< 0.01
Diagnosis (reference: psychosis)			
Non-psychotic disorders	−0.84 (1.05)	−0.79	0.42

### 3.4 Impact of the COVID-19 pandemic

Table 5 shows the perceived degree of impact of the COVID-19 pandemic on accessing services, perceived helpfulness of DHTs on accessing care during the pandemic, and willingness to use DHTs after the pandemic. Over a third (*n* = 170/447, 38.0%) of respondents reported the pandemic had no impact at all on their access care. Only a small proportion of respondents (*n* = 26/447, 5.8%) felt the pandemic had a significant impact for them, reporting either ‘moderately’ or ‘very much’. Most respondents considered DHTs could help them get access to mental health services during the pandemic (*n* = 260/447, 58.2%) and were willing to use such technologies to access mental health services after the pandemic (*n* = 266/447, 59.5%).

**Table 5:** The impact of COVID-19 pandemic regarding digital mental health.

Item	Not at all	Slightly	Somewhat	Moderately	Extremely
Has COVID-19 pandemic impacted on you being able to access a mental health service?	170 (38.0%)	159(35.6%)	92 (20.6%)	13 (2.9%)	13 (2.9%)
As the face-to-face visit to the hospital/clinic is limited, do you think digital technology can help you get access to mental health services during the COVID-19 pandemic?	74 (16.5%)	113 (25.3%)	193 (43.2%)	26 (5.8%)	41 (9.2%)
Would you be willing to use digital technology to access mental health services after the COVID-19 pandemic?	81 (18.1%)	100 (22.4%)	186 (41.6%)	17 (3.8%)	63 (14.1%)

## 4 Discussion

The study reports a survey on the usage of digital technologies and attitudes towards DHTs of people with SMI and explores the impact of the COVID-19 pandemic on their views of digital mental health in the Chinese context. To capture a diverse sample of people with SMI, the survey was distributed across different types of hospitals located in Beijing, China, including psychiatric hospitals, general hospitals with a psychiatric unit, secondary hospitals, and community healthcare centres. In total, 447 participants completed the survey.

We found that the ownership rates of digital technologies among people with SMI in China were high, with smartphones (95.5%), Internet (82.1%), and computers (72.7%) being the technologies that participants most commonly owned or had access to. These findings are in line with prior studies conducted in China whereby 90.9% of people with schizophrenia had access to the Internet (36) and 83.2% of people with mental health problems frequently used mobile devices (35). Moreover, the ownership rates of smartphones in people with SMI was comparable to the general population, which is 96% (19). Compared to results reported from other countries, the smartphone ownership rate found in this study is higher than an early meta-analysis of studies published from 2009 to 2015, which showed an average mobile phone ownership rate among people with psychosis being 66.4% (46). This is comparable with a more recent study which found the ownership rates of mobile phone and smartphone among people with psychosis in the UK was 95 and 90%, respectively (47). Furthermore, most of the respondents reported having no barriers to owning or using a mobile phone. These findings suggest that people with SMI in China had a high ownership rate of digital technologies and were able to use such technologies the same as in the general population, which paved the way for implementing DHTs in this population.

Although respondents reported a high ownership rate of digital technologies, several findings from the survey suggest potential challenges for the implementation of DHTs in people with SMI in China, including the low usage of health-related usage of digital technologies and health-related smartphone apps. One potential explanation for the discrepancy between the high ownership of digital technologies and low health-related usage might be related to the lack of awareness about DHTs. Similar challenges were also identified in other countries (48, 49), and programmes have been developed to improve digital health literacy and narrow the knowledge gap (49). For instance, the Digital Opportunities for Outcomes in Recovery Services (DOORS) is a group training programme developed in the US to help people with SMI improve their digital health literacy (50, 51). However, no similar programmes have been developed in China.

Undoubtedly, the COVID-19 pandemic had an unprecedented impact on mental healthcare provision (52, 53). Strikingly, nearly a quarter of respondents felt the pandemic caused no impact at all on their access to mental health services. This may be associated with the Chinese government’s rapid action in providing online mental health services right after lockdown (38). As expected, most respondents found DHTs being useful for access to mental health services during the pandemic and were willing to use DHTs after the pandemic. During the data collection period, Beijing was in the “dynamic zero COVID-19 case” stage. In this stage, the priority was to promptly detect infections and possible close contacts and massive lockdown strategies were replaced by region-wide static management (54). Therefore, the disruption of healthcare provision was milder compared to the first wave of COVID-19, and the mobility of people was not restricted if no positive COVID-19 case was detected in their residential area. In December 2022, the Chinese government lifted restrictions on COVID-19 (55), which means there will be no restrictions on face-to-face health services. A follow-up study is needed to explore the uptake of DHTs among people with SMI after in-person mental health services are resumed in China.

### 4.1 Limitations

There are some limitations in the current study. First, participants were only recruited from hospitals in Beijing. Although we attempted to diversify the types of hospitals, the sample still lacks national-level representativeness. Also, as Beijing is one of the economically developed cites in China, participants may have higher rate of access to digital technologies compared to other geographic regions in China. Second, because the survey did not collect data on which types of hospital respondents sought care in, we could not analyse the differences across hospital types. Future research may be needed to understand differences in ownership of digital technologies and views on DHTs of people with SMI from different healthcare institutes. Third, although we measured barriers participants faced to being able to own or use a mobile phone, the barriers to use a mobile phone and other digital technologies for mental health purpose remains unclear. Future studies, including qualitative work investigating barriers directly related to the usage of mobile phones and other digital technologies for mental health purpose among people with SMIs in China is needed. Finally, an online survey may overestimate the accessibility of digital technologies of participants since it requires participants to use a digital device to access the survey.

## 5 Conclusion

In sum, the current study found that people with SMI in China had a high ownership and usage rate of digital technologies. In contrast, respondents’ health-related usage of digital technologies was less frequent, and their attitudes toward DHTs were neutral. These findings suggest the need for training programmes to improve digital health literacy for people with SMI in China to fully realise the potential of DHTs.

## Data availability statement

The raw data supporting the conclusions of this article will be made available by the authors, without undue reservation.

## Ethics statement

The studies involving humans were approved by the ethics committee of the University of Manchester and Beijing Anding Hospital of Capital Medical University. The studies were conducted in accordance with the local legislation and institutional requirements. The participants provided their written informed consent to participate in this study.

## Author contributions

XZ: Conceptualization, Data curation, Formal Analysis, Investigation, Writing – original draft. SL: Conceptualization, Supervision, Writing – review & editing. XC: Investigation, Writing – review & editing. NB: Conceptualization, Writing – review & editing. SB: Conceptualization, Supervision, Writing – review & editing.
