# Supplementary material for: Technology use and attitudes towards digital mental health in people with severe mental health problems: a survey study in China
Source: Front Psychiatry. 2023 Nov 22;14:1261795. doi: 10.3389/fpsyt.2023.1261795 (PMC10702738; doi:10.3389/fpsyt.2023.1261795)
Supplement: Supplementary file 1 [file Data_Sheet_1.docx]

**Supplementary table 1.** The English version of the survey

**Study title:**

**Staff and service users views on implementing digital technology in mental health service in China: a mixed-methods study**

**Survey (service user version)**

**Version 1. 29/06/2020**

**Instruction**

**Welcome to the project**

We would like to invite you to participate in a survey focusing on digital mental health in China. As a person with experiences of mental health issues, your response to this survey is very valuable to us. We wish to collect your views on using digital technology to support and deliver mental health service in China. The questions concern your ownership and usage of digital technologies and your attitudes about using digital tools in the context of mental health delivery. We also would like to collect information about how the COVID-19 pandemic has changed your views on digital mental health. Your response to this survey, and those of everyone who decides to take part, will inform the future development of a digital mental health tool to help people with severe mental health problems.

In the survey, digital mental health is defined as applying digital technologies such as short message service (SMS) messages, mobile apps, computer programmes, software applications (apps), interactive websites, social media platforms, wearable and ambient sensors, virtual reality, or artificial intelligence to mental healthcare.

Thank you for participating in the survey. Your answers are very valuable to us. If you have questions about this project, please contact us: [xiaolong.zhang@postgrad.manchester.ac.uk](mailto:xiaolong.zhang@postgrad.manchester.ac.uk)

**Part I. Demographic information**

1. How old are you? ……..

2. What is your gender? ……………………

3. Where is your current location: ………………Province………………City

4. What are your living arrangements?

Lived alone Lived with others

5. What is your marital status?

Single Divorced

Married Widowed

6. What is the highest level of education you have completed? Please tick to indicate

High school or some high school University or some university

Postgraduate course

7. What is your employment status? Please tick to indicate:

Working full-time Student

Working part-time Currently unemployed

Self-employed

8. What is your monthly household income per person?

Under 1,000 RMB 10,000 – 15,000 RMB

1,000 – 5,000 RMB Above 15,000 RMB

5,000 – 10,000 RMB

9. What is your diagnosis? ………………………………………………..

**Part II. Ownership and usage of digital technology/digital mental health tools**

1. Which of the following do you own or have access to? (please circle to indicate):

| Mobile phone (non-smartphone) | Yes (I own one) | Yes (but it belongs to someone else) | No |
| --- | --- | --- | --- |
| Mobile phone (smartphone) | Yes (I own one) | Yes (but it belongs to someone else) | No |
| iPad or other types of tablet | Yes (I own one) | Yes (but it belongs to someone else) | No |
| Laptop or desktop computer | Yes (I own one) | Yes (but it belongs to someone else) | No |
| Wearable fitness tracker (e.g. Xiaomi Mi band, HUAWEI band) | Yes (I own one) | Yes (but it belongs to someone else) | No |
| Smartwatch (e.g. Apple watch) | Yes (I own one) | Yes (but it belongs to someone else) | No |
| Hearables/smart headphones (e.g. Apple AirPods) | Yes (I own one) | Yes (but it belongs to someone else) | No |
| Virtual reality headsets (e.g. Google Cardboard, Oculus Go) | Yes (I own one) | Yes (but it belongs to someone else) | No |
| Internet | Yes (I pay) | Yes (but someone else pays) | No |

2. What operating system does your smartphone use?

iOS (Apple iPhone) Android Don’t know

NA (I do not use a smartphone)

**3. What features do you use on your phone (please tick all that apply):**

Phone calls Alarm

Text messages Calendar

Email Radio/Podcast

Internet browser Music

Smartphone apps Camera

Built-in GPS (e.g. map, navigation) Gaming

Other……………………

4. How often do you use the following? (please circle to indicate)

| Mobile phone | Multiple times a day | Once a day | A few times a week | Once a week | A few times a month | Once a month | Less often | N/A I do not use this |
| --- | --- | --- | --- | --- | --- | --- | --- | --- |
| Smartphone | Multiple times a day | Once a day | A few times a week | Once a week | A few times a month | Once a month | Less often | N/A I do not use this |
| Smartphone apps | Multiple times a day | Once a day | A few times a week | Once a week | A few times a month | Once a month | Less often | N/A I do not use this |
| Internet via mobile phone/tablet | Multiple times a day | Once a day | A few times a week | Once a week | A few times a month | Once a month | Less often | N/A I do not use this |
| Internet via computer/laptop | Multiple times a day | Once a day | A few times a week | Once a week | A few times a month | Once a month | Less often | N/A I do not use this |
| Social media (e.g. WeChat, Weibo) | Multiple times a day | Once a day | A few times a week | Once a week | A few times a month | Once a month | Less often | N/A I do not use this |
| Smartwatch (e.g. Apple watch) | Multiple times a day | Once a day | A few times a week | Once a week | A few times a month | Once a month | Less often | N/A I do not use this |
| Fitness tracker (e.g. Xiaomi Mi band, HUAWEI band) | Multiple times a day | Once a day | A few times a week | Once a week | A few times a month | Once a month | Less often | N/A I do not use this |
| Hearables/smart headphones (e.g. Apple AirPods) | Multiple times a day | Once a day | A few times a week | Once a week | A few times a month | Once a month | Less often | N/A I do not use this |
| Laptop computer | Multiple times a day | Once a day | A few times a week | Once a week | A few times a month | Once a month | Less often | N/A I do not use this |
| Desktop computer | Multiple times a day | Once a day | A few times a week | Once a week | A few times a month | Once a month | Less often | N/A I do not use this |
| Tablet computer (e.g. iPad) | Multiple times a day | Once a day | A few times a week | Once a week | A few times a month | Once a month | Less often | N/A I do not use this |
| Virtual reality headsets (e.g. Google Cardboard, Oculus Go) | Multiple times a day | Once a day | A few times a week | Once a week | A few times a month | Once a month | Less often | N/A I do not use this |

5. Aside from telephone calls, how frequently do you use a computer, mobile phone, smartwatch， tablet computer or other digital technology to do the following? (please circle to indicate):

| Identify coping strategies | Very often | Often | Sometimes | Rarely | Never |
| --- | --- | --- | --- | --- | --- |
| Monitor symptoms | Very often | Often | Sometimes | Rarely | Never |
| Develop relationships with other individuals who have a lived experience related to mental health problems | Very often | Often | Sometimes | Rarely | Never |
| Provide support to others | Very often | Often | Sometimes | Rarely | Never |
| Set alarms/reminders to help with medication management | Very often | Often | Sometimes | Rarely | Never |
| Use calendar or set alerts/reminders for appointments | Very often | Often | Sometimes | Rarely | Never |
| Find information about mental health problems | Very often | Often | Sometimes | Rarely | Never |
| Find information about physical health problems | Very often | Often | Sometimes | Rarely | Never |
| Listen to music or audio files to help block or manage voices | Very often | Often | Sometimes | Rarely | Never |
| Record voices or sounds that I hear that others do not | Very often | Often | Sometimes | Rarely | Never |
| Take photos of objects or people that I see that others do not | Very often | Often | Sometimes | Rarely | Never |

6. How often do you use the following mobile phone apps? (please circle to indicate)

| Instant messaging apps (e.g. WeChat, QQ) | Multiple times a day | Once a day | A few times a week | Once a week | A few times a month | Once a month | Less often | I have downloaded this type of app, but never used it | I have never downloaded this type of app |
| --- | --- | --- | --- | --- | --- | --- | --- | --- | --- |
| Social media apps (e.g. WeChat moment, Weibo) | Multiple times a day | Once a day | A few times a week | Once a week | A few times a month | Once a month | Less often | I have downloaded this type of app, but never used it | I have never downloaded this type of app |
| Entertainment apps (e.g. TicTok, Youku Video, Bilibili) | Multiple times a day | Once a day | A few times a week | Once a week | A few times a month | Once a month | Less often | I have downloaded this type of app, but never used it | I have never downloaded this type of app |
| Video calling apps (e.g. FaceTime, Skype) | Multiple times a day | Once a day | A few times a week | Once a week | A few times a month | Once a month | Less often | I have downloaded this type of app, but never used it | I have never downloaded this type of app |
| Gaming apps (e.g. Brain Training, Candy Crush) | Multiple times a day | Once a day | A few times a week | Once a week | A few times a month | Once a month | Less often | I have downloaded this type of app, but never used it | I have never downloaded this type of app |
| Exercise apps (e.g. Keep) | Multiple times a day | Once a day | A few times a week | Once a week | A few times a month | Once a month | Less often | I have downloaded this type of app, but never used it | I have never downloaded this type of app |
| Diet/healthy eating apps (e.g. Change4Life, MyFitnessPal) | Multiple times a day | Once a day | A few times a week | Once a week | A few times a month | Once a month | Less often | I have downloaded this type of app, but never used it | I have never downloaded this type of app |
| Mindfulness/ meditation apps (e.g. Headspace) | Multiple times a day | Once a day | A few times a week | Once a week | A few times a month | Once a month | Less often | I have downloaded this type of app, but never used it | I have never downloaded this type of app |
| Mental health apps | Multiple times a day | Once a day | A few times a week | Once a week | A few times a month | Once a month | Less often | I have downloaded this type of app, but never used it | I have never downloaded this type of app |

7. If you have ever used mental health/wellbeing/mindfulness app(s), please can you indicate below which apps these were, whether you currently use them, how often you use them and how helpful you found them (1 = unhelpful; 4 = very helpful)? If you cannot remember the name of the app, perhaps check your phone or alternatively write a description of what the app does.

| App name | Current or past use *(circle to indicate)* | | Frequency of use (1=multiple times a day; 2=once a day; 3=a few times a week; 4=once a week; 5=a few times a month; 6=less often) | | | | | | Perceived helpfulness (1= unhelpful - it made me feel worse; 2 = neutral - it didn’t help but it didn’t make me feel worse; 3 = helpful; 4 = very helpful) | | | |
| --- | --- | --- | --- | --- | --- | --- | --- | --- | --- | --- | --- | --- |
| App 1 (please specify)  …………………………  ………………………… | Current | Past | 1 | 2 | 3 | 4 | 5 | 6 | 1 | 2 | 3 | 4 |
| App 2 (please specify)  …………………………  ………………………… | Current | Past | 1 | 2 | 3 | 4 | 5 | 6 | 1 | 2 | 3 | 4 |
| App 3 (please specify)  …………………………  ………………………… | Current | Past | 1 | 2 | 3 | 4 | 5 | 6 | 1 | 2 | 3 | 4 |
| App 4 (please specify)  …………………………  ………………………… | Current | Past | 1 | 2 | 3 | 4 | 5 | 6 | 1 | 2 | 3 | 4 |
| App 5 (please specify)  …………………………  ………………………… | Current | Past | 1 | 2 | 3 | 4 | 5 | 6 | 1 | 2 | 3 | 4 |

N/A (I have never used a mental health app)

8. What are the barriers you have faced, if any, to being able to own or use a mobile phone? (please tick all that apply):

I struggle to afford to own and/or use a mobile phone

I’m not interested in mobile phones

I don’t need to use a mobile phone

I keep losing or damaging mobile phones

I don’t know how to use a mobile phone

I don’t know how to use certain mobile phone features (e.g. smartphone apps)

I feel paranoid or suspicious about mobile phones

Not applicable (there are no barriers for me)

Other (please state) ………………………

9. Have you ever shared any of the following information with a member of your care team?

a. Information you have found online about psychiatric medications

Yes No

b. Information you have found online about psychological therapy

Yes No

10. Interest in future services.

10.1 Would you want to be able to access general information related to your health via your smartphone?

Yes No
10.2 Would you want to receive text messages on your phone related to your health from your doctor’s office?

Yes No
10.3 Would you want to use your phone to help track your medical condition via an application or “app” on your smartphone?

Yes No

10.4 Would you download an application or “app” to your phone to help monitor your health condition?

Yes No
10.5 Would you be willing to use an application or “app” on your phone on a daily basis to help monitor your health condition?

Yes No

10.6 Would you be willing to permit background data gathering of your phone usage (e.g. call logs, device activity, etc.) to collect data related to your health condition?

Yes No

10.7 Would you be willing to use the built-in sensors or GPS on your phone to monitor your health condition?

Yes No

10.8 Would you want to use an artificial agent (e.g. iPhone Siri, Baidu Xiaodu, Xiaomi Xiaoai) to help manage your health condition?

Yes No

**Part III. Attitudes**

**The following questions concern about your attitudes to digital health interventions (DHIs). Please circle the most appropriate score for each item.**

*****Digital health interventions are defined as applying digital technologies such as short message service (SMS) messages, mobile apps, computer programmes, software applications (apps), interactive websites, social media platforms, wearable and ambient sensors, virtual reality, or artificial intelligence to deliver or support mental healthcare.

|  | **Totally agree** | **Rather agree** | **Not sure** | **Rather disagree** | **Totally disagree** |
| --- | --- | --- | --- | --- | --- |
| By using a DHI, I do not expect long-term effectiveness | **1** | **2** | **3** | **4** | **5** |
| By using a DHI, I do not receive professional support | **1** | **2** | **3** | **4** | **5** |
| It is difficult to implement the suggestions of a DHI effectively in everyday life | **1** | **2** | **3** | **4** | **5** |
| DHIs could increase isolation and loneliness | **1** | **2** | **3** | **4** | **5** |
| A DHI can help me to recognize the issues that I have to challenge | **1** | **2** | **3** | **4** | **5** |
| I have the feeling that a DHI can help me | **1** | **2** | **3** | **4** | **5** |
| A DHI can inspire me to better approach my problems | **1** | **2** | **3** | **4** | **5** |
| I believe that the concept of DHIs makes sense | **1** | **2** | **3** | **4** | **5** |
| In crisis situations, a therapist can help me better than a DHI | **1** | **2** | **3** | **4** | **5** |
| I learn skills to better manage my everyday life from a therapist rather than from a DHI | **1** | **2** | **3** | **4** | **5** |
| I am more likely to stay motivated with a therapist than when using a DHI | **1** | **2** | **3** | **4** | **5** |
| I do not understand therapeutic concepts as well with a DHI as I do with a therapist | **1** | **2** | **3** | **4** | **5** |
| A DHI is more confidential and discreet than visiting a therapist | **1** | **2** | **3** | **4** | **5** |
| By using a DHI, I can reveal my feelings more easily than with a therapist | **1** | **2** | **3** | **4** | **5** |
| I would be more likely to tell my friends that I use a DHI than that I visit a therapist | **1** | **2** | **3** | **4** | **5** |
| By using a DHI, I do not have to fear that someone will find out that I have psychological problems | **1** | **2** | **3** | **4** | **5** |

**Part IV. COVID-19 pandemic**

1. Has COVID-19 pandemic impacted on you being able to access a mental health service?

Scale:
 0 =not at all 5 = very much

Not at all 0-------1-------2-------3-------4-------5 Very much

1. As the face-to-face visit to the hospital/clinic is limited, do you think digital technology can help you get access to mental health services during the COVID-19 pandemic?

Scale:
 0 =not at all 5 = very much

Not at all 0-------1-------2-------3-------4-------5 Very much

1. Would you be willing to use digital technology to access mental health services after the COVID-19 pandemic?

Scale:
 0 =not at all 5 = very much

Not at all 0-------1-------2-------3-------4-------5 Very much
