# Supplementary material for: Technology use and attitudes towards digital mental health in people with severe mental health problems: a survey study in China
Source: Front Psychiatry. 2023 Nov 22;14:1261795. doi: 10.3389/fpsyt.2023.1261795 (PMC10702738; doi:10.3389/fpsyt.2023.1261795)
Supplement: Supplementary file 2 [file Data_Sheet_2.doc]

**Supplementary table 2.** The Chinese version of the survey

**研究题目：**

**中国精神心理机构医务人员及患者对数字医疗技术临床应用的观点：一项混合方法研究**

**调查问卷（患者版）**

**版本1. 29/06/2020**

**介绍**

欢迎参与本研究

我们想邀请您参加一项针对中国数字化精神心理健康的调查。作为有精神心理健康问题经验的人，您对本调查的答复对我们非常有价值。我们希望收集您对在中国使用数字技术支持和进行精神心理健康服务的态度和观点。问卷中的问题将收集包括您拥有或使用数字技术的情况以及您对使用数字技术提供精神心理健康服务的态度的相关信息。我们还想收集有关新冠肺炎疫情如何影响了您对数字心理健康的看法。您以及所有决定参加本研究的人的回答，将为数字化精神心理健康工具的未来发展提供信息，从而为帮助患有严重精神健康问题的人群提供支持。

在调查中，数字心理健康被定义为应用数字技术，例如短信、移动应用程序（APP）、计算机程序、软件应用程序、交互式网站、社交媒体平台、可穿戴和环境传感器、虚拟现实技术或人工智能技术提供精神心理健康服务。

感谢您参与调查。您的回答对我们非常有价值。如果您对本研究有任何疑问，请联系我们：[xiaolong.zhang@postgrad.manchester.ac.uk](mailto:xiaolong.zhang@postgrad.manchester.ac.uk)

**第一部分：人口学信息**

1.你的年龄？ ………………

2.您的性别？ ………………

3.您的现居住地？………………省………………城市

4.您的居住情况？

□ 独居 □ 与他人同住

5. 您的婚姻状况？

□ 单身 □ 离异

□ 已婚 □ 丧偶

6.您的学历？

□ 高中或同等学历院校 □ 大学或同等学历院校

□ 研究生及以上

7.您的工作状态？ 请勾选以表示：

□ 全职工作 □ 学生

□ 兼职 □ 目前失业

□ 自由职业

8.您的家庭人均月收入？

□ 1000元以下 □ 10,000 – 15,000元

□ 1,000 – 5,000元 □ 15,000元以上

□ 5,000 – 10,000元

9.您目前患有的精神心理问题的诊断是什么？ ……………………………………..

**第二部分 数字技术/数字化精神心理健康工具的持有和使用情况**

1. 您拥有或可以使用以下哪些数字产品或数字技术？ （请在适合的选项上画圈）：

| 手机（非智能手机） | 是的（我拥有一个） | 是的（但它属于其他人） | 没有 |
| --- | --- | --- | --- |
| 手机（智能手机） | 是的（我拥有一个） | 是的（但它属于其他人） | 没有 |
| iPad平板电脑或其他类型的平板电脑 | 是的（我拥有一个） | 是的（但它属于其他人） | 没有 |
| 笔记本电脑或台式电脑 | 是的（我拥有一个） | 是的（但它属于其他人） | 没有 |
| 可穿戴运动手环（如小米或者华为手环） | 是的（我拥有一个） | 是的（但它属于其他人） | 没有 |
| 智能手表（如苹果手表） | 是的（我拥有一个） | 是的（但它属于其他人） | 没有 |
| 智能耳机（如苹果智能耳机AirPods） | 是的（我拥有一个） | 是的（但它属于其他人） | 没有 |
| VR虚拟设备（如 Google Cardboard, Oculus Go） | 是的（我拥有一个） | 是的（但它属于其他人） | 没有 |
| 互联网 | 是的 (我自己支付互联网费用) | 是的 (但由其他人为我支付互联网费用) | 没有 |

2．您的智能手机使用什么操作系统？

□ IOS苹果系统 □ 安卓系统 □ 不清楚

□ 不适用（我不使用智能手机）

3.您在手机上使用哪些功能（请勾选所有适用的功能）：

□ 电话 □ 闹钟

□ 短信 □ 日历

□ 电子邮件 □ 广播/播客

□ 互联网浏览器 □ 音乐

□ 智能手机应用软件 □ 相机

□ 内置GPS（例如地图，导航）游戏

□ 其他……

4.您多久使用一次以下内容？ （请圈出以表示）

| 移动电话 | 一天使用多次 | 一天使用一次 | 一周使用多次 | 一周使用一次 | 一月使用多次 | 每月使用一次 | 不经常使用 | 不适用（我不使用这个） |
| --- | --- | --- | --- | --- | --- | --- | --- | --- |
| 智能手机 | 一天使用多次 | 一天使用一次 | 一周使用多次 | 一周使用一次 | 一月使用多次 | 每月使用一次 | 不经常使用 | 不适用（我不使用这个） |
| 智能手机应用程序（APP） | 一天使用多次 | 一天使用一次 | 一周使用多次 | 一周使用一次 | 一月使用多次 | 每月使用一次 | 不经常使用 | 不适用（我不使用这个） |
| 通过手机/平板电脑上网 | 一天使用多次 | 一天使用一次 | 一周使用多次 | 一周使用一次 | 一月使用多次 | 每月使用一次 | 不经常使用 | 不适用（我不使用这个） |
| 通过电脑/笔记本电脑上网 | 一天使用多次 | 一天使用一次 | 一周使用多次 | 一周使用一次 | 一月使用多次 | 每月使用一次 | 不经常使用 | 不适用（我不使用这个） |
| 社交软件（如微信，微博） | 一天使用多次 | 一天使用一次 | 一周使用多次 | 一周使用一次 | 一月使用多次 | 每月使用一次 | 不经常使用 | 不适用（我不使用这个） |
| 智能手表（如苹果手表） | 一天使用多次 | 一天使用一次 | 一周使用多次 | 一周使用一次 | 一月使用多次 | 每月使用一次 | 不经常使用 | 不适用（我不使用这个） |
| 健身手环（如小米手环，华为手环） | 一天使用多次 | 一天使用一次 | 一周使用多次 | 一周使用一次 | 一月使用多次 | 每月使用一次 | 不经常使用 | 不适用（我不使用这个） |
| 智能耳机（如苹果耳机） | 一天使用多次 | 一天使用一次 | 一周使用多次 | 一周使用一次 | 一月使用多次 | 每月使用一次 | 不经常使用 | 不适用（我不使用这个） |
| 笔记本电脑 | 一天使用多次 | 一天使用一次 | 一周使用多次 | 一周使用一次 | 一月使用多次 | 每月使用一次 | 不经常使用 | 不适用（我不使用这个） |
| 台式电脑 | 一天使用多次 | 一天使用一次 | 一周使用多次 | 一周使用一次 | 一月使用多次 | 每月使用一次 | 不经常使用 | 不适用（我不使用这个） |
| 平板电脑（如苹果iPad平板电脑） | 一天使用多次 | 一天使用一次 | 一周使用多次 | 一周使用一次 | 一月使用多次 | 每月使用一次 | 不经常使用 | 不适用（我不使用这个） |
| VR虚拟设备（如Google Cardboard, Oculus Go） | 一天使用多次 | 一天使用一次 | 一周使用多次 | 一周使用一次 | 一月使用多次 | 每月使用一次 | 不经常使用 | 不适用（我不使用这个） |

5.除了打电话，您有多经常会使用计算机、手机、智能手表、平板电脑或其他数字技术完成以下事项？ （请在对应的选项上画圈）：

| 寻找应对方法 | 频繁使用 | 经常使用 | 有时使用 | 很少使用 | 从不使用 |
| --- | --- | --- | --- | --- | --- |
| 监测症状 | 频繁使用 | 经常使用 | 有时使用 | 很少使用 | 从不使用 |
| 与其他具有精神心理健康问题相关经历的人联系 | 频繁使用 | 经常使用 | 有时使用 | 很少使用 | 从不使用 |
| 为他人提供支持 | 频繁使用 | 经常使用 | 有时使用 | 很少使用 | 从不使用 |
| 设置闹钟/提醒以帮助进行药物管理 | 频繁使用 | 经常使用 | 有时使用 | 很少使用 | 从不使用 |
| 使用日历或设置闹钟/提醒就诊 | 频繁使用 | 经常使用 | 有时使用 | 很少使用 | 从不使用 |
| 查找有关精神心理健康问题的信息 | 频繁使用 | 经常使用 | 有时使用 | 很少使用 | 从不使用 |
| 查找有关身体健康问题的信息 | 频繁使用 | 经常使用 | 有时使用 | 很少使用 | 从不使用 |
| 收听音乐或其他音频文件以帮助阻止或管理幻听 | 频繁使用 | 经常使用 | 有时使用 | 很少使用 | 从不使用 |
| 录制我能听到但别人听不到声音 | 频繁使用 | 经常使用 | 有时使用 | 很少使用 | 从不使用 |
| 拍摄我能看到，但别人看不到的物体或人物的照片 | 频繁使用 | 经常使用 | 有时使用 | 很少使用 | 从不使用 |

6.您多久使用一次以下手机应用程序？ （请在适当的选项上画圈）

| 即时通讯应用程序  如微信，QQ | 一天多次 | 一天一次 | 一周几次 | 一周一次 | 一月多次 | 一月一次 | 不经常 | 我下载了应用程序，但未使用过 | 从未下载过这类应用程序 |
| --- | --- | --- | --- | --- | --- | --- | --- | --- | --- |
| 社交软件如微信，微博 | 一天多次 | 一天一次 | 一周几次 | 一周一次 | 一月多次 | 一月一次 | 不经常 | 我下载了应用程序，但未使用过 | 从未下载过这类应用程序 |
| 娱乐软件如TicTok，优酷视频，哔哩哔哩 | 一天多次 | 一天一次 | 一周几次 | 一周一次 | 一月多次 | 一月一次 | 不经常 | 我下载了应用程序，但未使用过 | 从未下载过这类应用程序 |
| 视频通话软件如FaceTime skype | 一天多次 | 一天一次 | 一周几次 | 一周一次 | 一月多次 | 一月一次 | 不经常 | 我下载了应用程序，但未使用过 | 从未下载过这类应用程序 |
| 游戏软件如脑力训练，糖果消消乐 | 一天多次 | 一天一次 | 一周几次 | 一周一次 | 一月多次 | 一月一次 | 不经常 | 我下载了应用程序，但未使用过 | 从未下载过这类应用程序 |
| 健身软件如keep | 一天多次 | 一天一次 | 一周几次 | 一周一次 | 一月多次 | 一月一次 | 不经常 | 我下载了应用程序，但未使用过 | 从未下载过这类应用程序 |
| 规律/健康饮食软件  如Change4Life, MyFitnessPal | 一天多次 | 一天一次 | 一周几次 | 一周一次 | 一月多次 | 一月一次 | 不经常 | 我下载了应用程序，但未使用过 | 从未下载过这类应用程序 |
| 正念/冥想软件（如顶空） | 一天多次 | 一天一次 | 一周几次 | 一周一次 | 一月多次 | 一月一次 | 不经常 | 我下载了应用程序，但未使用过 | 从未下载过这类应用程序 |
| 心理健康软件 | 一天多次 | 一天一次 | 一周几次 | 一周一次 | 一月多次 | 一月一次 | 不经常 | 我下载了应用程序，但未使用过 | 从未下载过这类应用程序 |

7.如果您曾经使用过精神心理健康或正念相关的应用程序，请在下表中对这些应用程序进行描述，包括是什么应用程序？您当前是否使用它们？您使用它们的频率以及发现它们对您的帮助程度如何（1 =无帮助； 4 =非常有帮助）？ 如果您不记得该应用程序的名称，则可以产看您的手机或写下有关这个应用程序功能的说明。

| 应用名称 | 现在或过去的使用情况（在适当的选项上画圈） | | 使用频率（1 =每天多次； 2 =一天一次； 3 =一周几次； 4 =一周一次； 5 =每月几次； 6 =较少使用） | | | | | | 感知到的帮助（1 =无帮助-使我感到更糟； 2 =中立-没帮助但没有使我感到更糟； 3 =有用； 4 =很有帮助） | | | |
| --- | --- | --- | --- | --- | --- | --- | --- | --- | --- | --- | --- | --- |
| 应用程序1（请指定）…………………………  ………………………… | 现在 | 过去 | 1 | 2 | 3 | 4 | 5 | 6 | 1 | 2 | 3 | 4 |
| 应用程序2（请指定）…………………………  ………………………… | 现在 | 过去 | 1 | 2 | 3 | 4 | 5 | 6 | 1 | 2 | 3 | 4 |
| 应用程序3（请指定）…………………………  ………………………… | 现在 | 过去 | 1 | 2 | 3 | 4 | 5 | 6 | 1 | 2 | 3 | 4 |
| 应用程序4（请指定）…………………………  ………………………… | 现在 | 过去 | 1 | 2 | 3 | 4 | 5 | 6 | 1 | 2 | 3 | 4 |
| 应用程序5（请指定）…………………………  ………………………… | 现在 | 过去 | 1 | 2 | 3 | 4 | 5 | 6 | 1 | 2 | 3 | 4 |

□ 不适用（我从未使用过精神心理健康应用程序）

8.您持有或者使用手机是否面临任何的困难？如果有的话，是什么困难？ （请勾选所有适用的选项）：

□ 我难以负担拥有和/或使用手机的费用

□ 我对手机不感兴趣

□ 我不需要用手机

□ 我不断丢失或损坏手机

□ 我不知道怎么用手机

□ 我不知道如何使用某些手机功能（例如，智能手机应用）

□ 我对手机感到偏执或怀疑

□ 不适用（对我来说没有困难）

□ 其他（请说明）…………………………

9.您是否曾经与您所接触的医务人员分享过以下任何信息？

a．您在网上找到的有关精神科药物的信息

□ 是 □ 否

b．您在网上找到的有关心理治疗的信息

□ 是 □ 否

10.对未来服务的兴趣。

10.1您是否希望通过智能手机获得与您的健康相关的一般信息？

□ 是 □ 否

10.2您是否希望通过手机接收医生发给您的与您的健康有关的短信？

□ 是 □ 否

10.3您是否希望通过智能手机上的应用程序来帮助记录您的病情？

□ 是 □ 否

10.4您是否希望下载手机应用程序以帮助您监测自己的健康状况？

□ 是 □ 否

10.5您是否愿意每天在手机上使用应用程序来帮助监测您的健康状况吗？

□ 是 □ 否

10.6您是否愿意允许收集手机使用情况的后台数据（例如通话记录，设备活动等）来收集与您的健康状况有关的数据？

□ 是 □ 否

10.7您是否愿意使用手机上的内置传感器或GPS来监测您的健康状况？

□ 是 □ 否

10.8您是否愿意使用人工智能技术（例如iPhone Siri，百度小度，小米小爱）来帮助您管理健康状况？

□ 是 □ 否

**第三部分 态度**

以下问题将要了解您对数字医疗干预技术（DHI）的态度。请在每个选项上最合适的分数上画圈。

*数字医疗干预技术（DHI）被定义为应用数字技术，短信，移动应用程序，计算机程序，软件程序，交互式网站，社交媒体平台，可穿戴和环境传感器，虚拟现实或人工智能 提供或支持精神心理健康服务。

|  | 完全同意 | 基本同意 | 不确定 | 基本不同意 | 完全不同意 |
| --- | --- | --- | --- | --- | --- |
| 我不认为使用数字医疗技术会给我带来长期的治疗效果 | 1 | 2 | 3 | 4 | 5 |
| 使用数字医疗技术让我感到无法获得专业支持 | 1 | 2 | 3 | 4 | 5 |
| 在日常生活中很难有效实施数字医疗的建议 | 1 | 2 | 3 | 4 | 5 |
| 使用数字医疗可能会增加孤独感 | 1 | 2 | 3 | 4 | 5 |
| 数字医疗可以帮助我认识到我必须解决的问题 | 1 | 2 | 3 | 4 | 5 |
| 我感觉数字医疗可以帮助我 | 1 | 2 | 3 | 4 | 5 |
| 数字医疗可以激励我更好地解决问题 | 1 | 2 | 3 | 4 | 5 |
| 我相信数字医疗的概念很有意义 | 1 | 2 | 3 | 4 | 5 |
| 在危机情况下，医生/治疗师比数字医疗更能帮助我 | 1 | 2 | 3 | 4 | 5 |
| 我跟愿意向医生/治疗师而不是数字医疗学习技能，以更好地管理自己的日常生活 | 1 | 2 | 3 | 4 | 5 |
| 与使用数字医疗相比，与医生/治疗师工作更有可能让我保持动力 | 1 | 2 | 3 | 4 | 5 |
| 我对数字医疗的治疗概念的了解不如对医生/治疗师所提供的治疗的了解 | 1 | 2 | 3 | 4 | 5 |
| 使用数字医疗与看医生/治疗师相比能更好的保护隐私 | 1 | 2 | 3 | 4 | 5 |
| 与医生/治疗师相比，通过使用数字医疗技术，我可以更轻松地表露我的感受 | 1 | 2 | 3 | 4 | 5 |
| 相对于告诉我的朋友我去看医生/治疗师，我更可能告诉我的朋友我在使用数字医疗技术 | 1 | 2 | 3 | 4 | 5 |
| 使用数字医疗，我不必担心别人会发现我有心理问题 | 1 | 2 | 3 | 4 | 5 |

**第四部分 新冠肺炎疫情**

1. 新冠肺炎疫情是否影响您获得精神心理健康的医疗服务？

评分：0 =完全不会5 =非常影响

完全没有0 ------- 1 ------- 2 ------- 3 ------- 4 ------- 5 非常影响

2.由于医院面对面就诊的限制，您是否认为数字技术可以在新冠肺炎疫情期间帮助您获得精神心理健康相关的医疗服务？

评分：0 =完全不能5 =非常赞同

完全不能0 ------- 1 ------- 2 ------- 3 ------- 4 ------- 5 非常赞同

3.在新冠肺炎疫情之后，您是否愿意通过使用数字技术获得精神心理健康的医疗服务吗？

评分：0 =完全不会5 =非常愿意

完全不会0 ------- 1 ------- 2 ------- 3 ------- 4 -------5 非常愿意
