# Supplementary material for: Technology use and attitudes towards digital mental health in people with severe mental health problems: a survey study in China
Source: Front Psychiatry. 2023 Nov 22;14:1261795. doi: 10.3389/fpsyt.2023.1261795 (PMC10702738; doi:10.3389/fpsyt.2023.1261795)
Supplement: Supplementary file 3 [file Data_Sheet_3.docx]

**Supplementary table 3.** List of reported mental health, wellbeing or mindfulness apps

| **Respondent ID** | **App name (Chinese)** | **App name (English)** | **Current or past use** | **Frequency of use** | **Perceived helpfulness** |
| --- | --- | --- | --- | --- | --- |
| 15 | 简单心理 | Jian Dan Xin Li | Current | A few times a month | Helpful |
| 70 | 北京尚善公益 | Beijing Shangshan Foundation | Current | A few times a week | Helpful |
| 97 | 呼吸 | Breath | Current | A few times a week | Helpful |
| 104 | 小睡眠 | CoSleep | Past | Less often | Unhelpful |
| 135 | 解忧娃娃 | Worrydolls | Current | Once a week | Helpful |
| 148 | 壹点心理 | Yidian Psychology | Past | Less often | Helpful |
| 148 | 简单心理 | Jian Dan Xin Li | Past | Less often | Helpful |
| 157 | 月食 | KnowYourself | Current | Once a day | Helpful |
| 189 | 壹心理 | One Psychology | Past | Less often | Neutral |
| 189 | 心理咨询壹点灵 | Yidianling Counselling | Past | Less often | Neutral |
| 189 | 知乎 | Zhihu | Current | A few times a week | Very helpful |
| 206 | 蜗牛睡眠 | Snail Sleep | Current | Once a day | Helpful |
| 211 | 阳光心理 | Sunshine Psychology | Past | Multiple times a day | Helpful |
| 298 | Keep | Keep | Current | A few times a week | Helpful |
| 366 | 武志红心理 | Wuzhihong Psychology | Current | Once a week | Helpful |
| 497 | Keep | Keep | Past | Less often | Neutral |
| 511 | 潮汐 | Tide | Past | Once a week | Helpful |
| 871 | 冥想星球 | Mindfulness Planet | Past | A few times a week | Helpful |
| 901 | 壹心理 | Yi Psychology | Past | A few times a week | Helpful |
| 901 | 简单心理 | Jian Dan Xin Li | Current | A few times a week | Helpful |
| 945 | 睿心 | Wiseheart | Past | Multiple times a day | Helpful |
